# Tobacco use and health insurance literacy among vulnerable populations: implications for health reform

**DOI:** 10.1186/s12913-017-2680-7

**Published:** 2017-11-15

**Authors:** Robert T. Braun, Yaniv Hanoch, Andrew J. Barnes

**Affiliations:** 1 Department of Health Behavior and Policy, Virginia Commonwealth University, 830 E. Main St., 9th Floor, Richmond, Virginia, 23219 USA; 2 Department of Psychology, Plymouth University, Plymouth, UK

**Keywords:** Tobacco, Smoking, Health literacy, Health insurance literacy, Insurance

## Abstract

**Background:**

Under the Affordable Care Act (ACA), millions of Americans have been enrolling in the health insurance marketplaces. Nearly 20% of them are tobacco users. As part of the ACA, tobacco users may face up to 50% higher premiums that are not eligible for tax credits. Tobacco users, along with the uninsured and racial/ethnic minorities targeted by ACA coverage expansions, are among those most likely to suffer from low health literacy – a key ingredient in the ability to understand, compare, choose, and use coverage, referred to as health insurance literacy. Whether tobacco users choose enough coverage in the marketplaces given their expected health care needs and are able to access health care services effectively is fundamentally related to understanding health insurance. However, no studies to date have examined this important relationship.

**Methods:**

Data were collected from 631 lower-income, minority, rural residents of Virginia. Health insurance literacy was assessed by asking four factual questions about the coverage options presented to them. Adjusted associations between tobacco use and health insurance literacy were tested using multivariate linear regression, controlling for numeracy, risk-taking, discount rates, health status, experiences with the health care system, and demographics.

**Results:**

Nearly one third (31%) of participants were current tobacco users, 80% were African American and 27% were uninsured. Average health insurance literacy across all participants was 2.0 (SD 1.1) out of a total possible score of 4. Current tobacco users had significantly lower HIL compared to non-users (−0.22, *p* < 0.05) after adjustment. Participants who were less educated, African American, and less numerate reported more difficulty understanding health insurance (*p* < 0.05 each.)

**Conclusions:**

Tobacco users face higher premiums for health coverage than non-users in the individual insurance marketplace. Our results suggest they may be less equipped to shop for plans that provide them with adequate out-of-pocket risk protection, thus placing greater financial burdens on them and potentially limiting access to tobacco cessation and treatment programs and other needed health services.

## Background

Under the Affordable Care Act (ACA), up to 14 million consumers were expected to gain health insurance through state and federal insurance marketplaces in 2016 [1]. Nearly 20% of these consumers were tobacco users who, prior to the ACA, were more likely to be uninsured [2, 3]. Importantly, under the ACA, tobacco users can face up to a 50% surcharge that is not eligible for income-based tax credits [4]. Recent evidence suggests low-socioeconomic status (SES) tobacco users are at particularly high risk of being unable to afford health care, and are reluctant to enroll in the marketplace, in part because of higher premiums due to the surcharge [4, 5].

Tobacco users, along with the uninsured and racial/ethnic minorities targeted by ACA coverage expansions, are among those most likely to suffer from low health literacy [6, 7]. Low-income and minority tobacco users who suffer from low health literacy are more likely to have nicotine dependence and more difficulty quitting, and are more likely to relapse after attempting to quit [8–11]. Such individuals are expected to benefit from coverage expansions through improved access to health care and cessation services. Notably, as this population engages with the marketplace to shop for a health plan, understanding the details and complexities of health insurance is essential. Health insurance literacy (HIL) and health literacy share common components. Both require an individual to understand, compare, and choose sufficient health services to meet their health care needs. However, HIL also measures an individual’s knowledge of the structure of health benefits and basic cost-sharing concepts [12]. This exploratory study was designed to examine the association between HIL and smoking among a largely low-SES, predominantly African American population.

Earlier data suggested that when consumers participated in a hypothetical scenario of shopping for health insurance—both novices and those with experience—lacked adequate knowledge about benefits and coverage [13], and beneficiaries frequently make poor insurance decisions [13, 14]. Insured individuals with low HIL tend to use their health coverage inefficiently (e.g., visiting the emergency room instead of urgent care), exposing them to excessive out-of-pocket spending risk [13]; they place more decision weight when comparing insurance plans on easier-to-understand features, like premiums, rather than more complex attributes like deductibles and out-of-pocket maximums. Also, low-HIL individuals are less likely to respond to incentives within their insurance policies (e.g., wellness incentives) [13] and they tend to utilize fewer adaptive decision strategies, instead often choosing health plans by word of mouth and not re-evaluating their coverage choices when health or financial circumstances change [13, 15].

In one study, less than a third of respondents gave correct answers to four questions about basic features of their insurance coverage, and overestimated insurance coverage restrictions, especially for approval of a specialist [16]. Other evidence suggests that individuals with private insurance understood their insurance-covered, in-patient services, but underestimated their insurance policy’s restrictions for outpatient services and prescription drugs [17]. In a third study, only 23% of respondents understood the terminology used in their health insurance policy, roughly 50% knew their monthly premium, and very few understood acronyms commonly used in health insurance [18]. Further, 48% of the adult population lacks the literacy and numeracy skills required to understand health information fully [19]. This may be exacerbated even more for tobacco users, because of their tendencies to be greater risk-takers and to be more present-oriented (i.e., higher discount rates of time) [13, 20–23]. This suggests that tobacco users may be willing to bear more out-of-pocket spending risk and focus more on immediate costs (e.g., premiums) rather than future costs (e.g., future out-of-pocket spending). Therefore, they may prefer plans with lower premiums that offer less coverage and expose them to higher out-of-pocket risk. Additionally, tobacco users suffer from lower levels of numeric literacy, or numeracy, and tend to be less educated, making it much more difficult to understand heath information [24]. Since tobacco users are more likely to be uninsured, they may also be less familiar with the terminology of insurance and how it works [13, 16]. Finally, evidence suggests that when tobacco users seek health coverage, they are less likely to enroll, which limits their access to tobacco cessation programs [5].

Given the body of evidence above, it is imperative to understand whether tobacco users are at a particular disadvantage and are less able to benefit from coverage expansions due to limited HIL so as to target enrollment and re-enrollment communication strategies better and to increase insurance uptake among tobacco users. This exploratory study examines the associations between current tobacco use and HIL among a vulnerable sample of rural, low-income, predominantly African American participants.

## Methods

A community sample of 647 rural Virginia residents was recruited using flyers posted in libraries and clinics, and through community recruiters. Individuals who were under the age of 65 were enrolled in the study and asked to complete an insurance choice experiment and survey at a local public library. A computer-based experiment was conducted using a hypothetical insurance marketplace like the one consumers face in 28 states including Virginia using the ACA’s federally facilitated marketplaces [25, 26]. Importantly, the experiment was incentive-compatible such that participant compensation was aligned with performance on the insurance choice task. Specifically, at the beginning of the study, participants were given 10,000 Monopoly dollars in each of two sequential choice tasks (Year 1 and Year 2) and told that each 100 Monopoly dollars is worth one real dollar. Participants were given two scenarios (Year 1 and Year 2) with a probability that they would fall ill and need to use health care services (e.g., 33% chance of getting sick having to go to the doctor 4 times in Year 1, and 80% chance of getting sick and having to go to the emergency room and then the hospital for several nights in Year 2 if they became sick in Year 1). After participants made their plan choices they were informed whether they were randomly assigned as healthy or sick, and the costs of premiums (or tax penalties if participants elected not to purchase insurance) and health care services, if sick, were deducted from their endowment of Monopoly dollars. Participants were paid a $5 show-up fee, up to $100 for the choice experiment (the average payout for Year 1 and Year 2) depending on their insurance decisions and chance, and $5 for completing a survey.

After the experiment, participants were asked to complete a survey that consisted of the following sections: health insurance literacy, tobacco use, cognitive function, risk-taking and discount rates, whether they purchased coverage in the insurance marketplace, demographics, health insurance, and health care experiences. While the main study assessed whether insurance plan recommendations affected plan choices using a hypothetical choice experiment [25], the analyses conducted below used data from the survey portion of the study to examine whether tobacco users reported lower health insurance literacy compared to non-users, after accounting for a number of potentially important common determinants of tobacco use and HIL, in order to inform policymakers about the potential unintended consequences of charging tobacco users more in the ACA marketplaces. A total of 631 participants with complete survey data comprised the study sample.

### Health insurance literacy

HIL was measured using an objective assessment of health insurance knowledge and skills [25, 26]. Participants viewed a table containing information in regards to six health care plans (Table 1), and were asked four HIL questions: (1) Which plan had the lowest deductible?; (2) Which plan had the highest out-of-pocket maximum?; (3) Which plan would be the lowest cost plan if no health services were needed in a year?; and (4) Which plan would be the lowest cost plan if $10,000 in health services were needed in a year?. HIL outcomes included correct responses to each question and a summary score of correct responses across all four HIL questions [26]. This objective assessment of HIL is similar to the items assessing the health insurance knowledge and skills domain used in previous studies [12]. The measure used in this study has been found in prior work to predict the ability of individuals to align health insurance choices with their preferences (i.e., to pick a plan with a low deductible when indicating that low deductibles are important to their plan choice) and to choose a plan that minimized total expected annual health care costs [26].

**Table 1:** Insurance Choice Task

Plan Name	A	B	C	D	E	F	No Insurance
Annual Premium	$108	$156	$400	$492	$1148	$1348	$695
Annual Deductible	$5500	$4500	$2250	$3350	$1000	$2000	Not applicable
OOP Maximum for the year	$6350	$6500	$6350	$5500	$3000	$3000	Not applicable
Primary Doctor Copay	$40	$35	$35	$45	$30	$30	100%
Cost of a 30 day supply of generic Rx	$20	$20	$15	$15	$15	$15	100%

Prompt: The ACA (also known as Obamacare) came into effect in November 2013. Obamacare offers uninsured individuals the opportunity to buy insurance. It is possible that you have already bought a health insurance plan. Regardless of whether you have insurance or not, please review the information presented in the next few pages and choose the insurance plan that you would pick if the decision were in real life. All insurance plans are for individual coverage. That is, imagine buying insurance for yourself. The table below presents information about six different health insurance plans. Please imagine your decision is a real one and that plan you choose will be the one you have for the next 12 months

### Tobacco use

Current tobacco users were defined as participants who had smoked over 100 cigarettes and now smoked cigarettes every day or some days [2]. A binary tobacco use variable was created to indicate current tobacco use.

### Numeracy

Numeracy was assessed using four items consisting of basic probability calculations from the Lipkus scale (e.g., Imagine that we are throwing a five-sided die 50 times. On average, out of these 50 throws how many times would this five-sided die show an odd number (1, 3, or 5)? [26, 27]. Extant evidence suggests that numeracy is a key determinant in comprehending and evaluating health insurance plans. Furthermore, researchers have also shown that numeracy is an independent element, distinct from education and intelligence that affects health insurance decision-making [26, 28, 29].

### Risk-taking

Risk-taking was assessed using the gambling domain of the Domain-Specific Risk-Taking (DOSPERT) Scale [30]. The risk-taking score was the average of responses on a seven-point Likert-type scale to three questions about how likely respondents were to engage in various financial activities (e.g., betting a day’s income at the horse races).

### Discount rates

Discount rates were evaluated to assess how present-oriented vs. future oriented participants were using four questions about preferences for winning and losing various amounts of money now vs. a year from now (i.e., win $20 vs. $30, lose $20 vs. $30, win $1000 vs. $1500, lose $1000 vs. $1500) [31]. Individuals who reported they would rather win less money now and lose more money later were considered to have higher discount rates (i.e., more present-oriented) and thus higher scores on this 0–4 scale.

### Demographics, health insurance, insurance marketplace, and health care experiences

Data were also gathered on participants’ age, gender, race, education, marital status, insurance status, and past year health care utilization history. Prior health care use was defined as a binary variable (“high-utilizer”) to indicate whether a participant had one or more inpatient admissions or two or more emergency room visits in the past year. This measure is consistent with prior literature and is derived from questions within the 2009 Medical Expenditures Panel Survey (MEPS) [26]. Additionally, data were gathered on whether an individual within the study purchased a health plan through the insurance marketplace.

### Statistical analysis

All analyses were conducted using Stata 13.1. Unpaired *t* tests and Chi-square tests were used to test for unadjusted associations between HIL, tobacco use, and other participant characteristics. We were also interested in the relationship between tobacco use and HIL after controlling for demographics, purchase coverage through the marketplace, numeracy, cognitive ability, risk taking, discount rates, and health care utilization. To understand the association between tobacco use and HIL better, we ran five adjusted models. The first four were linear probability models of the adjusted association between tobacco use and whether participants correctly comprehended characteristics of a simulated ACA marketplace plan and were able to identify the lowest deductible plan (Model 1), the plan with the highest out-of-pocket cost (Model 2), the plan with the lowest annual cost if the participant did not need any health care in a year (Model 3), and the plan with the lowest annual cost if the participant knew for certain he or she would be hospitalized and the cost of the procedure would be $10,000 (Model 4). Model 5 used ordinary least squares to estimate the adjusted association between tobacco use and the sum of correct responses to the four HIL questions. In all regression models, robust standard errors were used [32].

## Results

### Descriptive statistics

Among the 631 participants, nearly one third (31.5%) of respondents were current tobacco users and more than one in four (26.8%) were uninsured (Table 2). Participants were, on average, 40.2 years old (SD = 14.0). The sample was predominantly African American (80.0%) and female (68.6%), with roughly half having a high-school education or less (48.0%) and having never been married (49.0%). Participants scored low, on average, on the numeracy measure (0.5 [SD = 0.8] out of 4 possible correct questions). On average, half of the four HIL questions were answered correctly (M = 2.0, SD = 1.1). The majority of participants were able to identify the plan with the lowest deductible (63.6%) and highest out-of-pocket maximum (63.9%) but fewer than half could identify the lowest cost plan if no health services would be needed (46.0%) or if hospitalized and $10,000 in health services would be needed in a year (22.2%, for reference, the probability of guessing the answer to one HIL question correctly is one in seven, or 14%, and the probability of guessing all four correctly is less than 1%).

**Table 2:** Tobacco Use and Health Insurance Literacy (*n* = 631)

	Total Sample Characteristics	Tobacco Users	Non-Tobacco Users
	Mean or Percentage	Mean or Percentage	Mean or Percentage
	(SD)	(SD)	(SD)
Health insurance literacy (range 0–4)***	2.0	2.0	1.8
	(1.1)	(1.1)	(1.1)
Lowest Deductible (correct answer)*	63.6%	58.8%	65.7%
Highest OOP^a^ (correct answer)*	63.9%	59.3%	66.0%
Lowest Cost Choice if Health Services Not Needed (correct answer)	46.0%	44.2%	46.8%
Lowest Cost Choice if Hospitalized (correct answer)***	22.2%	15.1%	25.5%
Insurance Marketplace***	18.4%	12.6%	21.1%
Current Tobacco Use	31.5%	–	–
Risk Taking (range 1–7)***	1.9	2.1	1.7
	(1.2)	(1.2)	(1.2)
Discount Rate (range 0–4)	2.1	2.0	2.1
	(1.4)	(1.4)	(1.4)
Cognitive Function (range 0–1)	0.1	0.1	0.1
	(0.3)	(0.3)	(0.2)
Numeracy (range 0–4)	0.5	0.5	0.5
	(0.8)	(0.8)	(0.8)
Demographics			
Age***	40.2	36.8	42.5
	(14.0)	(12.3)	(14.4)
Race/Ethnicity			
African American	80.0%	80.9%	79.6%
White Non-Hispanic	17.3%	16.6%	17.6%
Other	2.7%	2.5%	2.8%
Female	68.6%	55.3%	74.8%
Marital Status**			
Never Married	49.0%	55.8%	45.8%
Widowed/Divorced/Separated	26.0%	24.6%	26.6%
Married	25.0%	19.6%	27.6%
Education***			
Less than High School	7.5%	11.8%	36.6%
High School	40.5%	49.0%	5.5%
Some College	37.2%	30.7%	40.2%
Bachelor’s/Graduate	14.8%	8.4%	17.7%
Type of Health Insurance***			
Employer-Sponsored	30.1%	18.6%	35.4%
Individual	10.9%	4.5%	13.9%
Medicare	10.8%	10.1%	11.1%
Medicaid	21.4%	24.12%	20.4%
Uninsured	26.8%	42.7%	19.4%
High Utilizers (> 1 ED visit or >0 inpatient admissions in the past year)***	35.4%	47.2%	30.8%
Monthly Income***			
$0–$720	26.3%	36.7%	21.5%
$721–$1200	26.0%	26.1%	25.9%
$1201–$2400	21.7%	23.1%	21.1%
$2401	26.0%	14.1%	31.5%
Heath Status			
Excellent	16.0%	12.6%	17.6%
Very Good	28.4%	27.6%	28.7%
Good	33.1%	33.2%	33.1%
Fair	19.5%	22.6%	18.1%
Poor	3.0%	4.0%	2.6%

*p < .10, **p < .05, ***p < .01

^a^Out-of-pocket (OOP) is the expenses for medical care that aren’t reimbursed by insurance. OOP costs include deductibles, coinsurance, and copayments for covered services plus all costs for services that aren’t covered

### Unadjusted associations between tobacco use, HIL, and participant characteristics

Participants who currently used tobacco were less likely to answer three of the four HIL questions correctly compared to those who were not current tobacco users. Specifically, tobacco users had more difficulty correctly identifying the insurance plan with the lowest deductible plan (58.8% vs. 66.0%, *p* < 0.10). The same pattern can be observed for identifying the plan with highest out-of-pocket costs (59.3% vs. 66.0%, p < 0.10), and the lowest cost plan if they were to be hospitalized (15.1% vs. 25.5%, *p* < 0.01). Consequently, tobacco users on average scored significantly lower on HIL than non-users (1.8 vs. 2.0, *p* < 0.01) before adjustment. Fewer tobacco users purchased a plan in the insurance marketplace than non-tobacco users (12.6% vs. 21.1%, p < 0.01). Tobacco users also had significantly higher risk-taking scores than non-users (2.1 vs. 1.7, p < 0.01) and had lower monthly income (p < 0.01). Moreover, age, marital status, education, type of health insurance, and health utilization varied significantly with current tobacco use (*p* < 0.05 each).

### Adjusted associations between HIL, tobacco use, and participant characteristics

Broadly speaking, current tobacco use was negatively associated with HIL after adjustment for demographics, purchase coverage through the marketplace, numeracy, cognitive ability, risk taking, discount rates, and health care utilization (Table 3). For example, tobacco users had an eight-percentage-point lower probability of correctly identifying the plan with the lowest annual cost if they were to become hospitalized (*p* < 0.05) and a nine-percentage-point lower probability of identifying the plan with the highest out-of-pocket costs (*p* < 0.10). However, tobacco users did not report any significant difference in identifying the plan with the lowest deductible or the plan with lowest annual cost if they knew for certain they would not get sick. In the model summarizing responses to the four HIL questions, tobacco use was associated with a 0.22-point decrease in HIL (*p* < 0.05).

**Table 3:** Marketplace and HIL

Variables	Lowest Deductible	Highest OOP^a^	Lowest Cost Choice if Health Services Not Needed	Lowest Cost Choice if Hospitalized	HIL
Tobacco Use					
Current Tobacco User	−0.07 (0.05)	−0.09* (0.05)	0.01 (0.05)	−0.08** (0.04)	−0.22** (0.10)
Insurance Market					
Purchase in Marketplace	−0.11* (0.05)	0.16*** (0.05)	0.02 (0.06)	0.02 (0.05)	−0.23** (0.12)
Demographics					
Age	−0.00** (0.00)	0.01*** (0.00)	0.00 (0.00)	0.00 (0.00)	−0.01** (0.00)
Female	0.06 (0.05)	0.04 (0.05)	−0.02 (0.05)	0.06 (0.04)	0.14 (0.10)
Race					
African-American (ref)	- -	- -	- -	- -	- -
White Non-Hispanic	0.10* (0.05)	0.04 (0.05)	0.09 (0.06)	0.04 (0.05)	0.27** (0.11)
Other	0.03 (0.13)	0.15* (0.08)	−0.03 (0.13)	0.12 (0.10)	0.27 (0.25)
Education					
Less than High School	−0.05 (0.08)	0.10 (0.08)	−0.11 (0.08)	−0.10** (0.05)	−0.16 (0.16)
High School (Ref)	- -	- -	- -	- -	- -
Some College	0.12*** (0.05)	0.06 (0.05)	0.06 (0.05)	0.03 (0.04)	0.27*** (0.10)
Bachelors/Graduate	0.03 (0.07)	−0.01 (0.06)	0.10 (0.06)	0.01 (0.05)	0.12 (0.14)
Marital Status					
Married (ref)	- -	- -	- -	- -	- -
Widowed/Divorced/Separated	0.13** (0.05)	0.08 (0.05)	−0.03 (0.06)	0.06 (0.05)	0.24** (0.12)
Married	0.06 (0.06)	−0.04 (0.06)	−0.11* (0.06)	0.01 (0.05)	−0.08 (0.12)
Type of Health Insurance					
Employer-sponsored (ref)	- -	- -	- -	- -	- -
Family Plan	0.00 (0.06)	0.09* (0.06)	0.05 (0.06)	0.00 (0.05)	0.14 (0.13)
Individual	−0.02 (0.08)	0.01 (0.08)	0.02 (0.08)	−0.06 (0.06)	−0.05 (0.16)
Medicare	0.10 (0.07)	0.06 (0.07)	−0.01 (0.08)	0.00 (0.06)	0.15 (0.17)
Medicaid	−0.05 (0.06)	−0.06 (0.06)	−0.01 (0.06)	−0.06 (0.05)	−0.18 (0.12)
Cognitive					
Cognitive Function	0.00 (0.07)	0.04 (0.07)	−0.06 (0.08)	0.18** (0.08)	0.17 (0.16)
Numeracy	0.06** (0.03)	0.08*** (0.02)	0.05* (0.03)	0.08*** (0.02)	0.25*** (0.06)
Risk					
Gambling Risk	0.02 (0.02)	0.02 (0.02)	0.01 (0.02)	−0.01 (0.01)	0.04 (0.04)
Discounting					
Impatience	0.00 (0.01)	0.01 (0.01)	−0.01 (0.01)	−0.01 (0.01)	0 (0.03)
Health					
High Utilizer	0.01 (0.04)	−0.06 (0.04)	0.08* (0.05)	0.01 (0.03)	0.03 (0.09)
Monthly Income					
$0–$720 (ref)	- -	- -	- -	- -	- -
$721–$1200	0.01 (0.05)	0.07 (0.05)	0.07 (0.06)	0.00 (0.04)	0.15 (0.11)
$1201–$2400	0.03 (0.06)	0.08 (0.06)	0.01 (0.06)	0.03 (0.05)	0.14 (0.13)
$2401	0.06 (0.07)	−0.00 (0.06)	0.13** (0.07)	0.02 (0.05)	0.21 (0.14)
Heath Status					
Excellent (ref)	- -	- -	- -	- -	- -
Very Good	0.10* (0.06)	0.10 (0.06)	0.03 (0.07)	0.09* (0.05)	0.33** (0.14)
Good	0.12* (0.06)	0.08 (0.06)	0.02 (0.06)	0.04 (0.05)	0.26** (0.13)
Fair	0.18** (0.07)	0.03 (0.07)	−0.05 (0.07)	0.05 (0.06)	0.21 (0.16)
Poor	0.10 (0.13)	−0.01 (0.12)	0.00 (0.13)	−0.02 (0.09)	0.07 (0.10)
Constant	0.49*** (0.11)	0.66*** (0.10)	0.32*** (0.11)	0.10 (0.08)	1.58*** (0.23)
Observations	631	631	631	631	631.00
R-squared	0.09	0.11	0.06	0.10	0.16

*** p < 0.01, ** p < 0.05, * p < 0.1

The linear regression models used robust standard errors

^a^Out-of-pocket (OOP) is the expenses for medical care that aren’t reimbursed by insurance. OOP costs include deductibles, coinsurance, and copayments for covered services plus all costs for services that aren’t covered

Many of the participant descriptors associated with disparities in health literacy and insurance coverage that are targeted by ACA coverage expansions had similar negative associations with HIL as tobacco use and were of similar magnitude. For example, compared to African American participants, White non-Hispanic participants were more likely to identify the plan with the lowest deductible (10 percentage points, *p* < 0.10) and have higher scores on the summary HIL measure (0.27-point increase, *p* < 0.05). Likewise, participants with some college compared to a high-school education were more likely to identify the plan with the lowest deductible and have higher scores on the summary HIL measure (*p* < 0.05 each). Further, more numerate participants had higher levels of HIL, having between a 5 and 8 percentage point increase in the adjusted probability of correctly answering individual HIL questions and 0.25 point increase in overall HIL per additional numeracy question answered correctly (p < 0.05 each). Risk-taking, discount rates, insurance status, marital status, education, income, and being a high utilizer of healthcare were not associated with HIL. However, individuals who purchased a health plan in the 2014 ACA insurance marketplace had more difficulty correctly identifying the plans with the highest out-of-pocket costs (−0.16, *p* < 0.01) and reported lower summary HIL scores (0.23, *p* < 0.05).

## Discussion

As of 2016, over 9 million people signed up for the ACA via the exchanges [34]. Of these 12 million, about 513,000 are reported tobacco smokers [34]. Tobacco users’ ability to navigate and decide which health insurance plan has enormous financial and health implications. Our study was specifically designed to examine this fundamental and important question, by creating a realistic (though hypothetical and simplified), exchange environment. We are concerned with health insurance literacy, given its key role in understanding health insurance information [8, 10, 11, 33–35]. For tobacco users, having low HIL may have long-term implications, because it suggests they are more likely to have difficulty anticipating their health care needs and selecting a health insurance plan that provides them with adequate risk protection. The ACA is expanding coverage to four million tobacco users, many of whom were previously uninsured [2, 3]. An unsubsidized surcharge of up to 50% on premiums was placed on tobacco users to internalize the voluntary risk-taking behavior that resulted in increased healthcare costs, increased consumption of health services, and, consequently, increased premiums [4, 36]. By construction, this surcharge raises the premium prices tobacco users face, having an unintended consequence of deterring them from purchasing insurance. Thus, the tobacco use surcharge could, paradoxically, impede health insurance uptake for vulnerable populations ultimately reducing opportunities for tobacco users to access much-needed health services, including tobacco cessation programs.

Our novel study demonstrates a robust inverse association between tobacco use and health insurance literacy. This evidence suggests that when tobacco users seek out a marketplace plan they may be essentially penalized twice: first, tobacco users are charged up to 50% more for a plan and may not be able to afford a plan based on health needs, exposing them to excess risk of out-of-pocket expenditures; second, they may have lower HIL and thus have more difficulty choosing coverage, creating additional barriers to accessing timely and needed care. Our results also highlight equivalence between more traditional indicators of health disparities and tobacco use. Notably, the gap in HIL between tobacco users and non-users, African American and White participants, those with a high school education and those with some college, and those with lower levels of numeracy compared to more numerate participants were similar in size. The link between numeracy and HIL among tobacco users [4, 36] further substantiates the importance of numeric literacy in understanding health insurance information [29]. Indeed, our results show that numeracy plays a greater role in HIL than risk-taking or time discounting, two traits that are commonly thought to be associated with tobacco use [26, 29, 37].

Our study is not without limitations. First, our sample is not representative, and thus it is unclear how generalizable our results are beyond our sample. However, we purposefully focused on a sample of rural, uninsured, mostly African American participants, as these populations are particularly important to ACA coverage expansion policies. We also included a broad array of controls for potential individual-level confounders to account for the influences of differences between the populations of tobacco users more broadly and those we studied. Second, it was outside the scope of this study to compare the role of HIL in the actual insurance plans selected by tobacco users and how these choices affected access to care and health and financial outcomes. Moreover, unlike Paez et al.’s HIL measure we did not measure document literacy or information seeking behaviors. However, both Paez et al.’s and our HIL measures assessed the health insurance knowledge and skills domain [12]. At the same time, it is likely that our results are very much conservative by nature, given our simplified choice environment. Indeed, participants in our study had to evaluate only six insurance plans, while the average number of plans on the exchanges is around 30 [38]. Furthermore, the six insurance plans were presented on a single page and in an easier format compare to the ones they are likely to view on the exchanges at the time of the study. Thus, it is possible that tobacco users ability to decide on an insurance plans is even further hampered when done on the exchanges. In regard to tobacco use, we only asked participants about their cigarette use but not about other types of tobacco products that may be common among a rural, predominantly African American sample (e.g., cigarillos or little cigars). Thus, we likely underrepresented the tobacco users in our sample and our results for cigarette users may not generalize to other types of tobacco users. Lastly, this study does not attempt to address whether or not smokers are less likely to shop for and enroll in coverage in private insurance marketplaces. It has been reported elsewhere that tobacco users make up a smaller proportion of health insurance marketplace enrollees than expected, suggesting that either tobacco users are indeed purchasing marketplace coverage but are savvy enough to misreport their tobacco use to avoid paying the surcharge, or, more likely, because tobacco users are more likely to have low health insurance literacy, they face greater challenges enrolling in marketplace coverage [34, 39].

## Conclusions

Tobacco users face higher premiums for health coverage than non-users in the individual insurance marketplace. Our results suggest that tobacco users may be less equipped to shop for plans that provide them with adequate out-of-pocket risk protection, thus placing greater financial burdens on them and potentially limiting access to tobacco cessation and treatment programs and other needed health services.

Ultimately, the success of health insurance marketplaces is predicated on consumers, including tobacco users, understanding the products being offered and actively participating in enrollment and re-enrollment. When there is a mismatch between health care needs and plan choices resulting from poor health insurance literacy, costs are often shifted to the safety net and other providers of uncompensated care. The difficulty many vulnerable populations face in choosing and using health insurance cast doubt on whether private insurance markets are managed and regulated sufficiently to achieve the aims of coverage expansion efforts. Importantly, our results lend preliminary evidence supporting tobacco use as a unique marker for low HIL among low-SES, minority populations and of potential importance to tailoring communication strategies aimed at mitigating health insurance coverage and access disparities populations with HIL. It also raises the importance of creating choice environments that facilitate the decision process within the exchanges. A number of studies have shown, for example, that providing consumers with their estimated total annual cost improves their choices [25, 26]. To address the complexity of selecting an insurance plan, Healthcare.gov added total cost calculators to aid prospective consumers in selecting plans under various health care utilization scenarios. Although, recent experimental evidence suggests such advances in choice architecture still leave pervasive gaps in insurance choice quality, particularly among vulnerable populations [40]. Drawing on the choice architecture literature could help alter the design of the insurance marketplace and, importantly, allow tobacco users’ to pick an insurance plan. This is especially important for smokers who are given the putative relationship between tobacco use and HIL. We encourage future studies to examine barriers to enrollment and access to explore the role of tobacco use in the ability to understand, compare, choose and use coverage. While further investigation is needed, policymakers should be cognizant of the link between tobacco use and HIL and should consider educational campaigns to help tobacco users, and indeed other vulnerable populations that tend to intersect with tobacco use, to select plans that provide them with adequate risk protection to reduce the financial burden on them, the safety net, and other providers of uncompensated care. Improving HIL among tobacco users may be critical to improving the quality of their insurance choices and the ability of coverage expansion policies in the ACA to reduce cancer burdens by improving access to tobacco cessation and cancer treatment.

## Abbreviations

ACA: Affordable care act
DOSPERT: Domain-specific risk-taking
HIL: Health insurance literacy
MEPS: Medical expenditure panel survey
SES: Socioeconomic status

## References

[1] How many individuals might have marketplace coverage at the end of 2016? 2015. https://aspe.hhs.gov/basic-report/how-many-individuals-might-have-marketplace-coverage-at-the-end-of-2016. Accessed 7 Feb 2016.

[2] Centers for Disease Prevention and Control. Current cigarette smoking among adults in the United States. 2013. http://www.cdc.gov/tobacco/data_statistics/fact_sheets/adult_data/cig_smoking/. Accessed 8 Feb 2016.

[3] Rheault M, McGeeney K. In the U.S., health insurance linked to better health habits. 2011. http://www.gallup.com/poll/151820/health-insurance-linked-better-health-habits.aspx. Accessed 9 Feb 2016.

[4] Giovannelli J, Lucia K, Corlette S. Insurance Premium Surcharges for Smokers May Jeopardize Access to Coverage. Commonwealth Fund. 2015:January 6, 2015; doi: http://www.commonwealthfund.org/publications/blog/2015/jan/insurance-premium-surcharges-for-tobacco-use. Accessed 7 Feb 2016.

[5] Friedman AS, Schpero WL, Busch SH (2016). Evidence suggests that the ACA’s tobacco surcharges reduced insurance take-up and did not increase smoking cessation. Health Affairs (Project Hope).

[6] KFF. Key facts about the uninsured population. 2015. http://kff.org/uninsured/fact-sheet/key-facts-about-the-uninsured-population/. Accessed 21 Feb 2016.

[7] Kutner M, Greenburg E, Jin Y, Paulsen, C. The health literacy of America’s adults: results from the 2003 national assessment of adult literacy. NCES 2006–483. Washington: National Center for Education Statistics; 2006.

[8] Arnold CL, Davis TC, Berke HJ, Jackson RH, Nandy I, London S. Smoking status, reading level, and knowledge of tobacco effects among low-income pregnant women. Prev Med. 2001;32:313–20. ​https://doi.org/10.1006/pmed.2000.0815. 10.1006/pmed.2000.0815 11304092

[9] Hoover DS, Vidrine JI, Shete S, Spears CA, Cano MA, Correa-Fernández V (2015). Health literacy, smoking, and health indicators in African American adults. J Health Commun.

[10] Stewart DW, Adams CE, Cano MA, Correa-Fernández V, Li Y, Waters AJ (2013). Associations between health literacy and established predictors of smoking cessation. Am J Public Health.

[11] Stewart DW, Cano MA, Correa-Fernandez V, Spears CA, Li Y, Waters AJ (2014). Lower health literacy predicts smoking relapse among racially/ethnically diverse smokers with low socioeconomic status. BMC Public Health.

[12] Paez KA, Mallery CJ, Noel H, Pugliese C, McSorley VE, Lucado JL (2014). Development of the health insurance literacy measure (HILM): conceptualizing and measuring consumer ability to choose and use private health insurance. J Health Commun.

[13] Loewenstein G, Friedman JY, McGill B, Ahmad S, Linck S, Sinkula S (2013). Consumers’ misunderstanding of health insurance. J Health Econ.

[14] Gabaix X, Laibson D. Shrouded attributes, consumer myopia, and information suppression in competitive markets. Q J Econ. 2006;121(2):505-40. doi: 10.3386/w11755.

[15] Frank RG, Lamiraud K (2009). Choice, price competition and complexity in markets for health insurance. J Econ Behav Organ.

[16] Cunningham PJ, Denk C, Sinclair M (2001). Do consumers know how their health plan works?. Health Affairs (Project Hope).

[17] Marquis MS (1983). Consumers’ knowledge about their health insurance coverage. Health Care Financing Review.

[18] Regence Group. Regence survey shows steep health plan learning curve. 2008. ​http://news.regence.com/releases/regence-survey-shows-steep-health-plan-learning-curve. Accessed 4 Apr 2017.

[19] Health literacy and America’s health insurance plans: laying the foundation and beyond. 2013. https://www.ahip.org/health-literacy-and-americas-health-insurance-plans-laying-the-foundation-and-beyond/. Accessed 15 Mar 2017.

[20] Bani M, Andorn A, Heidbreder C (2014). Pharmacologically, are smokers the same as non-smokers?. Curr Opin Pharmacol.

[21] Bickel WK, Odum AL, Madden GJ (1999). Impulsivity and cigarette smoking: delay discounting in current, never, and ex-smokers. Psychopharmacology.

[22] Hanoch Y, Johnson JG, Wilke A (2006). Domain specificity in experimental measures and participant recruitment: an application to risk-taking behavior. Psychol Sci.

[23] Kassel JD, Shiffman S, Gnys MA, Paty J, Zettler-Segal M (1994). Psychosocial and personality differences in chippers and regular smokers. Addict Behav.

[24] Martin LT, Haas A, Schonlau M, Derose KP, Rosenfeld L, Rudd R, et al. Which literacy skills are associated with smoking? J Epidemiol Community Health. 2012;66(2):189–92. 10.1136/jech.2011.136341. 10.1136/jech.2011.136341 PMC3246123 22003080

[25] Barnes AJ, Hanoch Y, Rice T (2016). Can plan recommendations improve the coverage decisions of vulnerable populations in health insurance marketplaces?. PLoS One.

[26] Barnes AJ, Hanoch Y, Rice T (2015). Determinants of coverage decisions in health insurance marketplaces: consumers’ decision making abilities and the amount of information in their choice environment. Health Serv Res.

[27] Lipkus IM, Samsa G, Rimer BK (2001). General performance on a numeracy scale among highly educated samples. Medical Decision Making: An International Journal of the Society for Medical Decision Making.

[28] Hibbard JH, Jewett JJ, Engelmann S, Tusler M (1998). Can Medicare beneficiaries make informed choices?. Health Affairs (Project Hope).

[29] Wood S, Hanoch Y, Barnes A, Liu P, Cummings J, Bhattacharya C (2011). Numeracy and Medicare part D: the importance of choice and literacy for numbers in optimizing decision making for Medicare’s prescription drug program. Psychol Aging.

[30] Weber EU, Blais A, Betz NE (2002). A domain specific risk attitude scale: measuring risk perceptions and risk behaviors. J Behav Decis Mak.

[31] Khwaja A, Silverman D, Sloan F (2007). Time preference, time discounting, and smoking decisions. J Health Econ.

[32] StataCorp L (2005). Stata base reference manual: Citeseer.

[33] Berkman ND, Sheridan SL, Donahue KE, Halpern DJ, Viera A, Crotty K, et al. Health literacy interventions and outcomes: an updated systematic review. Agency for Healthcare Research and Quality; 2011;1-E006:199.

[34] Pesko MF, Maclean JC, Kaplan CM, Hill SC. Trends over time in enrollment in non-group health insurance plans by tobacco use in the United States. Preventive Medicine Reports. London; 2017. 10.1016/j.pmedr.2017.05.010 PMC5447394 28593122

[35] Ramirez A, Westcombe A, Burgess C, Sutton S, Littlejohns P, Richards M. Factors predicting delayed presentation of symptomatic breast cancer: a systematic review. Lancet. 1999;353(9159):1127–31. doi: http://dx.doi.org/10.1016/S0140-6736(99)02142-X. 10.1016/s0140-6736(99)02142-x 10209975

[36] Kaplan CM, Graetz I, Waters TM (2014). Most exchange plans charge lower tobacco surcharges than allowed, but many tobacco users lack affordable coverage. Health Affairs (Project Hope).

[37] Hanoch Y, Rice T, Cummings J, Wood S (2009). How much choice is too much? The case of the Medicare prescription drug benefit. Health Serv Res.

[38] Avery K, Gardner M, Gee E, Marchetti-Bowick E, McDowell A, Sen A. Health plan choice and premiums in the 2016 Health Insurance Marketplace.Office of the Assistant Secretary for Planning and Evaluation, US Department of Health and Human Services. 2016.

[39] Liber AC, Hockenberry JM, Gaydos LM, Lipscomb J. The potential and peril of health insurance tobacco surcharge programs: evidence from Georgia’s state employees’ health benefit plan. Nicotine Tobacco Res. 2013; https://doi.org/10.1093/ntr/ntt216. 10.1093/ntr/ntt216 24376279

[40] Barnes AJ, Hanoch Y, Rice T, Long SK. Moving beyond blind men and elephants: providing total estimated annual costs improves health insurance decision making. Med Care Res Rev. 2016; ​https://doi.org/10.1177/1077558716669210. 10.1177/1077558716669210 27624636

